# Identification of key pathways, genes and immune cell infiltration in hypoxia of high-altitude acclimatization *via* meta-analysis and integrated bioinformatics analysis

**DOI:** 10.3389/fgene.2023.1055372

**Published:** 2023-03-24

**Authors:** Qiong Li, Zhichao Xu, Fujin Fang, Yan Shen, Huan Lei, Xiaobing Shen

**Affiliations:** ^1^ Key Laboratory of Environmental Medicine Engineering, Ministry of Education, School of Public Health, Southeast University, Nanjing, Jiangsu, China; ^2^ Department of Epidemiology and Health Statistics, School of Public Health, Southeast University, Nanjing, Jiangsu, China

**Keywords:** high-altitude acclimatization, meta-analysis, bioinformatics analysis, immune infiltration, hypoxia

## Abstract

**Background:** For individuals acutely exposed to high-altitude regions, environmental hypobaric hypoxia induces several physiological or pathological responses, especially immune dysfunction. Therefore, hypoxia is a potentially life-threatening factor, which has closely related to high-altitude acclimatization. However, its specific molecular mechanism is still unclear.

**Methods:** The four expression profiles about hypoxia and high altitude were downloaded from the Gene Expression Omnibus database in this study. Meta-analysis of GEO datasets was performed by NetworkAnalyst online tool. Kyoto Encyclopedia of Genes and Genomes (KEGG), Gene ontology (GO) enrichment analysis, and visualization were performed using R (version 4.1.3) software, respectively. The CIBERSORT analysis was conducted on GSE46480 to examine immune cell infiltration. In addition, we experimentally verified the bioinformatics analysis with qRT-PCR.

**Results:** The meta-analysis identified 358 differentially expressed genes (DEGs), with 209 upregulated and 149 downregulated. DEGs were mostly enriched in biological processes and pathways associated with hypoxia acclimatization at high altitudes, according to both GO and KEGG enrichment analyses. ERH, VBP1, BINP3L, TOMM5, PSMA4, and POLR2K were identified by taking intersections of the DEGs between meta-analysis and GSE46480 and verified by qRT-PCR experiments, which were inextricably linked to hypoxia. Immune infiltration analysis showed significant differences in immune cells between samples at sea level and high altitudes.

**Conclusion:** Identifying the DEGs and pathways will improve our understanding of immune function during high-altitude hypoxia at a molecular level. Targeting hypoxia-sensitive pathways in immune cells is interesting in treating high-altitude sickness. This study provides support for further research on high-altitude acclimatization.

## 1 Introduction

The change in immune function caused by hypoxia is of mounting medical and public concern worldwide. The supply of oxygen, which varies in different tissues, has been demonstrated to be vital for the correct functioning of all organs and systems in the human body. Hypoxia occurs due to the restriction of oxygen supply, leading to significant changes in oxygen-dependent physiological processes (Knox et al., 2017). Severe hypoxia has been associated with disease outbreaks and has also long been known to impact immune function (Taylor and Colgan, 2017).

One study has shown that hypoxia affects immune cell survival, while immune cells also have important roles in hypoxia homeostasis (Monaci et al., 2020). Hypoxia drives cell maturation in immunologic niches, accompanying inflammation in peripheral tissues (Norris et al., 2019). And it was confirmed that hypoxia is often correlated with macrophage content, neovessel count, and the expression of the hypoxia-inducible transcription factor 1α (HIF-1α) (Mateo et al., 2014). Furthermore, activation of HIFs and the HIF-1 pathway are critical in regulating early high-altitude acclimatization and participating in the pathogenesis of un-acclimatization (Schweizer et al., 2019).

**FIGURE 1:**
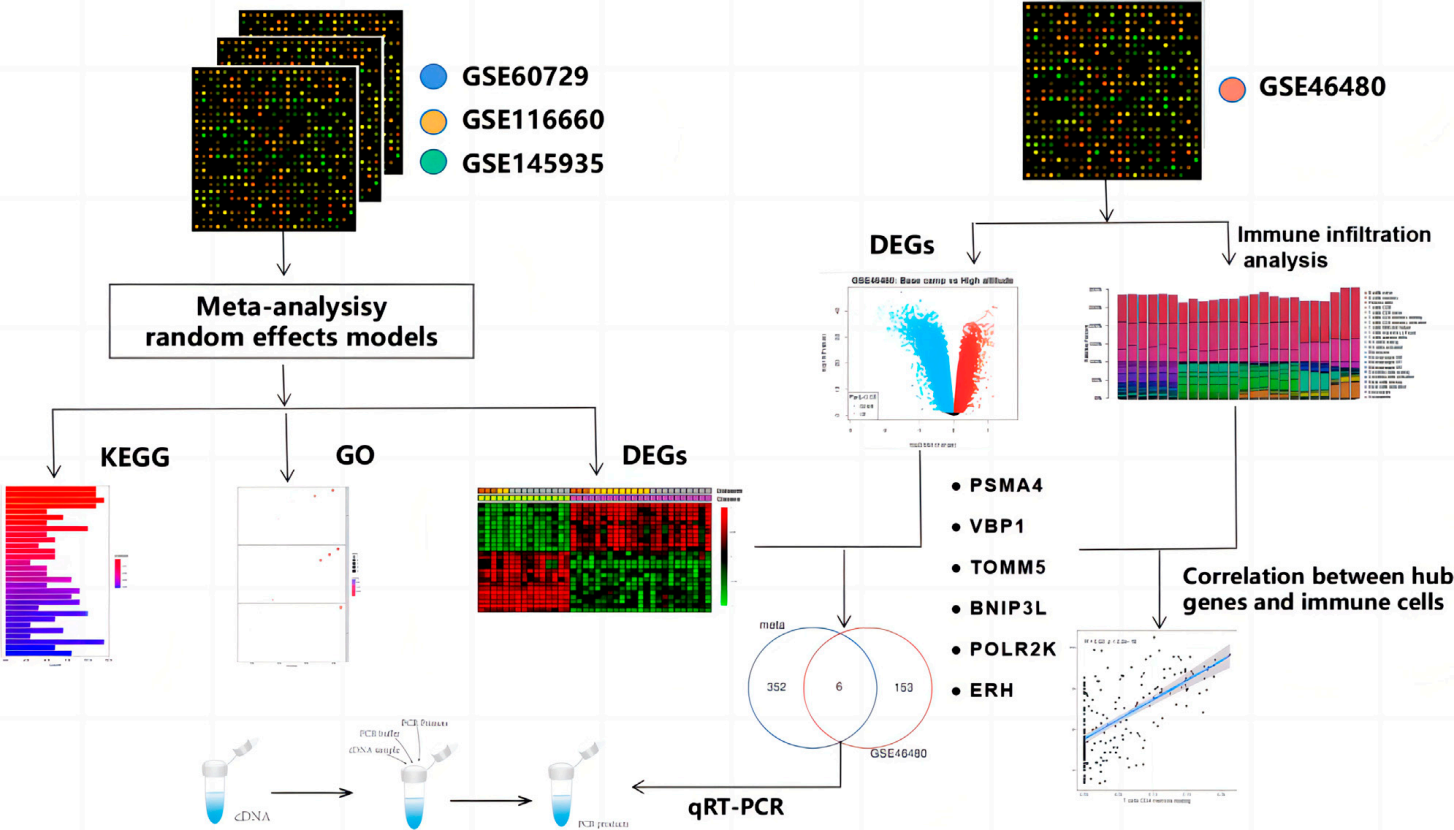
Workflow of this study.

High-altitude acclimatization can be defined as a physiological process in our body upon exposure to hypobaric hypoxia. It comprises a series of responses in different body systems, including restoring, transporting, and utilizing oxygen (O^2^) (Liu et al., 2016). Acute exposure to high altitude is associated with a spectrum of disorders encompassing acute mountain sickness (AMS), high altitude pulmonary edema (HAPE), and high altitude cerebral edema (HACE) in un-acclimatized individuals (Duan et al., 2020). Additionally, high-altitude hypoxia can also interfere with a variety of physiological functions, including immune function (Mei et al., 2020). Thus, studying high-altitude acclimatization is important to protect the health of people traveling and working at high altitudes. Here, we aim to access the molecular mechanism of the effect of high-altitude acclimatization on immune function. But, *in vivo* data on immunomodulatory effects of hypoxia or hypoxia mimetics in animal models are conflicting (Kiers et al., 2018). Meta-analysis of gene expression data sets was performed to help identify potential molecular signatures and gain insights into underlying biological processes based on gene expression datasets. The combination of meta-analysis and bioinformatics analyses may help to resolve existing inconsistencies and contradictions. We wanted to investigate whether there are common key genes that play important roles in multiple types of cells and influence immune function. To verify this hypothesis, we performed a systematic review of transcriptomics datasets from different cells in hypoxia and normoxia in the Gene Expression Omnibus (GEO), followed by consistent processing of meta-analysis, pathway enrichment, and overlap analyses. Then we download GSE46480 as the validation dataset for meta-analysis and immune infiltration analysis. At last, we experimentally verified the bioinformatics analysis with quantitative reverse-transcription polymerase chain reaction (qRT-PCR). This study aimed to demonstrate a systematic workflow for evidence synthesis of transcriptomic studies using meta-analysis and bioinformatics methods to identify potential biomarkers and pathogenic factors during high-altitude acclimatization. Here, we present a workflow of this study, as shown in Figure 1.

## 2 Materials and methods

### 2.1 Database search and datasets selection

**FIGURE 2:**
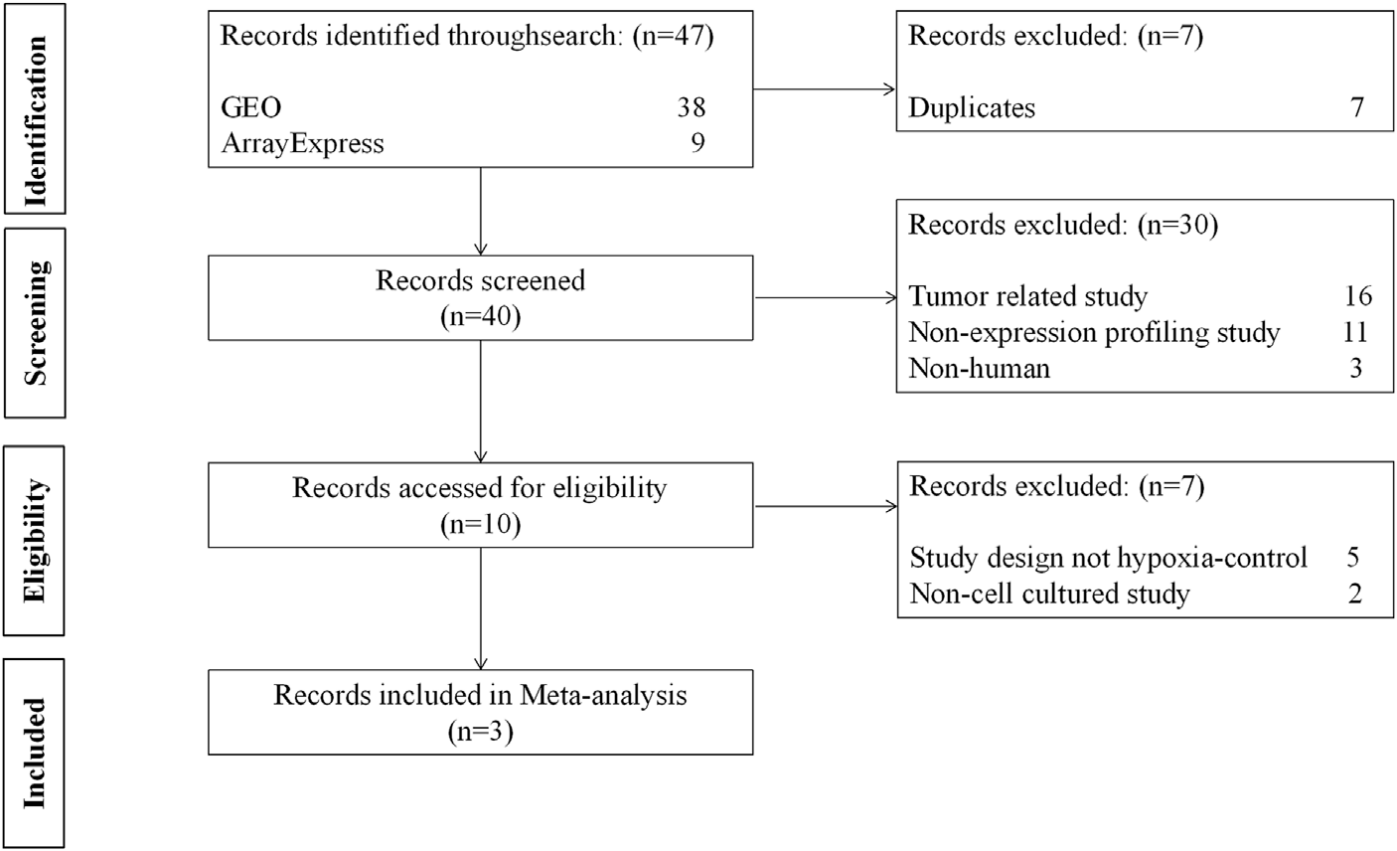
The flow diagram of datasets selection, including identification, screening, eligibility and inclusion stage.

We inquired about the two databases on 1 March 2022, for meta-analysis: GEO from the National Center for Biotechnology Information (NCBI) (Barrett et al., 2013) and ArrayExpress from the European Bioinformatics Institute (EMBL-EBI) (Athar et al., 2019). The search strategy is shown in Supplementary File S1 and the keywords used in the search were hypoxia and immune. Eligible datasets were those meeting all inclusion criteria and none of the exclusion criteria (Figure 2). The datasets were excluded if they were: 1) A tumor-related study; 2) A non-expression profiling study; 3) Not human cells; 4) A study design other than hypoxia/control; 5) Not cell culture. Finally, GSE145935, GSE116660, and GSE60729 were selected for meta-analysis, which included 12 normoxia samples and 12 hypoxia samples. Then, we choose GSE46480 as the validation dataset for immune infiltration analysis, which compares healthy volunteers at sea level with those exposed to altitude acutely. The details of the four GEO datasets are shown in Supplementary File S2.

### 2.2 Data normalization, meta-analysis and identification of DEGs

The online tool, NetworkAnalyst (http://www.networkanalyst.ca) (Zhou et al., 2019), performs a meta-analysis on three GEO datasets to normalize the data and determine the differentially expressed genes (DEGs). The merged data of NetworkAnalyst was shown in Supplementary File S3.

**FIGURE 3:**
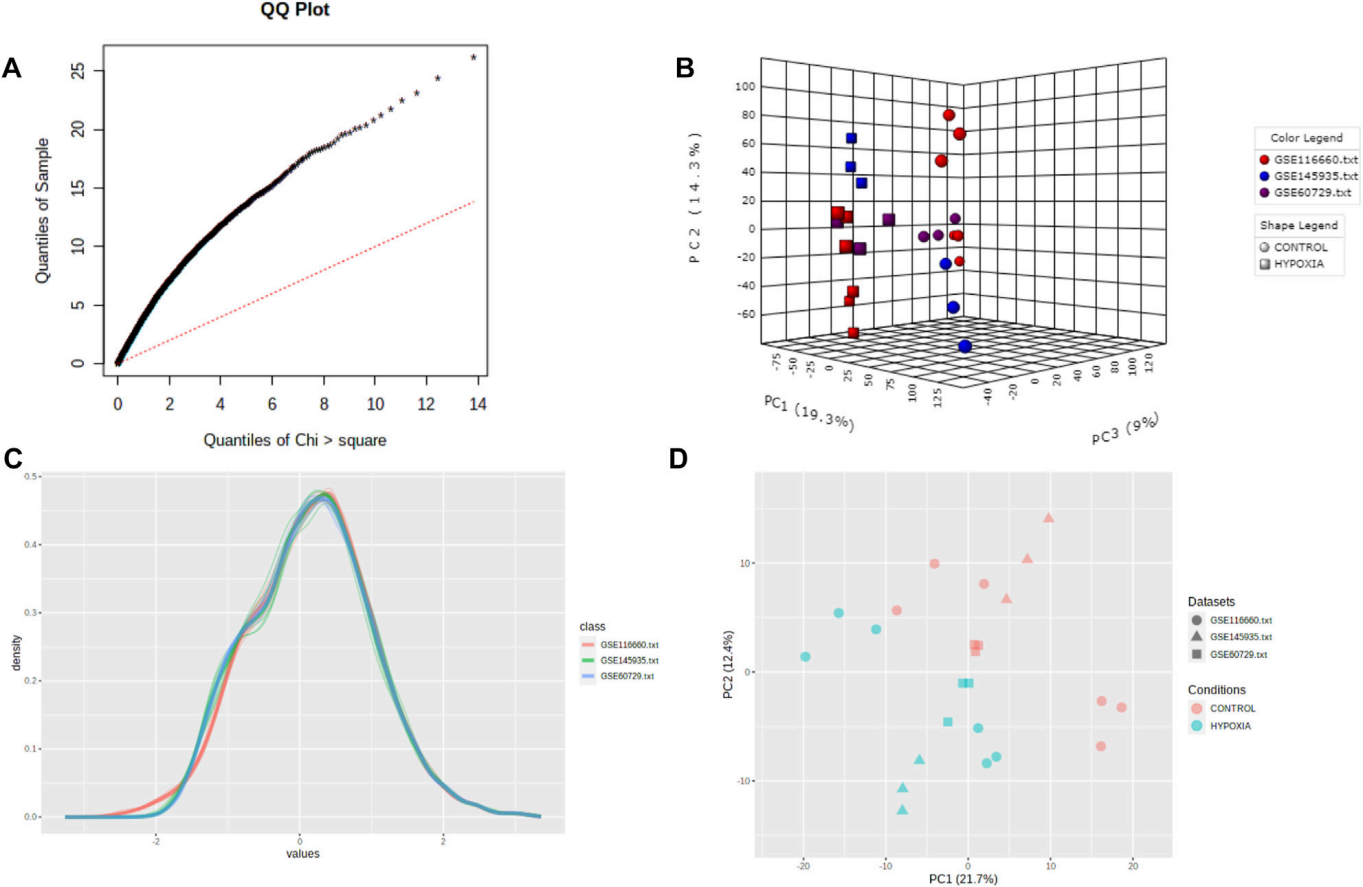
Quality control of meta-analysis. **(A)** The QQ-plot of Cochran’s Q test. **(B)** The three-dimensional plot of PCA score. **(C)** The density plot of the datasets. **(D)** The plane figure of PCA.

As a result of the batch-effect adjustment, a random-effects model (REM) was used in Combining Effect Sizes for the meta-analysis. The effect size is defined as the difference between two group means divided by standard deviation, considered combinable and comparable across different studies. The REM model can also incorporate unknown cross-study heterogeneities through a random effect. We selected REM based on statistical heterogeneity estimated using Cochran’s Q test (Figure 3A). Cochran’s Q test is calculated as the weighted sum of squared differences between individual study effects and the pooled effect across studies. It suggests that fixed-effects models (FEMs) should be assumed when the estimated Q values have an approximately chi-squared distribution. The use of REM is usually recommended if the distribution deviates significantly from a chi-squared distribution.

The GEO2R online tool (http://www.ncbi.nlm.nih.gov/geo/geo2r/) provided by the NCBI GEO database enabled comparisons between healthy participants at sea level and those exposed to altitude for the GSE46480 dataset.

All of the differential expression analysis is based on the “limma” method. Benjamini–Hochberg’s False Discovery Rate (FDR) is used to adjust the cut-off *p*-values. The genes were considered DEGs between normoxia and hypoxia samples when they had an FDR < 0.05 and a log (|FC|) > 1. To better visualize these DEGs, we made heatmaps and volcano plots by using NetworkAnalyst software and GEO2R.

### 2.3 Biological enrichment analysis

After the meta-analysis, a biological enrichment analysis was conducted on the statistically significant DEGs. Kyoto Encyclopedia of Genes and Genomes (KEGG), Gene Ontology (GO) enrichment analysis and visualization were performed using the packages: “colorspace”, “stringi”, “DOSE”, “clusterProfiler”, “digest”, “GOplot”, “ggplot2” of R software (version 4.1.3). Our analysis focused on the terms associated with the three main GO categories: biological processes, molecular functions, and cellular components. Q < 0.05 was used to select the significantly enriched functions and pathways. Q is the adjusted *p*-value.

### 2.4 Immune infiltration by CIBERSORT analysis

We performed the relative percent and content of 22 immune cell subsets by using CIBERSORT analytical tool (Newman et al., 2019). Using the “corrplot” package, we calculated the percentage of immune cells in the gene expression matrix and the relationship between genes and immune cells. After that, “vioplot” package was used to visualize CIBERSORT results.

### 2.5 Quantitative reverse-transcription polymerase chain reaction (qRT-PCR)

We used a lung epithelial cell line (BEAS-2B cells) and a human monocytic leukemia cell line (THP 1 cells) in culture media [RPMI medium 1,640 supplemented with 10% fetal bovine serum (Invitrogen)] for the validation of the mRNA expression between control and hypoxia groups. Cells in control groups were cultured 24 h under 5% CO_2_, 21% O_2_, and atmospheric pressure, while cells in hypoxia groups were cultured 24 h under 5% CO_2_, 1% O_2_, and 65 kPa for air pressure. Total RNA was extracted from the BEAS-2B cells and THP 1 cells by using RNA-easy Isolation Reagent (Vazyme, Nanjing, China). RNA was reverse-transcribed into complementary DNA (cDNA) using StarScript ш All-in-one RT Mix with gDNA Remover (GenStar, Beijing, China). qRT-PCR was performed by 2 × RealStar Green Fast Mixture with ROX (GenStar, Beijing, China). The primers were synthesized by The Beijing Genomics Institute (BGI) (Beijing, China), as described in Supplementary File S4. The fold change in relative mRNA expression was calculated using the 2^−ΔΔCt^ method.

### 2.6 Statistics analysis

The R software (4.1.3 version) was used to conduct statistical analyses. The Student’s t-test and Kruskal–Wallis test were used to compare the differences between the two groups. As a result, *p* < 0.05 (*), *p* < 0.01 (**), *p* < 0.001 (***), and *p* < 0.0001 (****) were considered significant.

## 3 Results

### 3.1 Identification of DEGs based on meta-analysis

**FIGURE 4:**
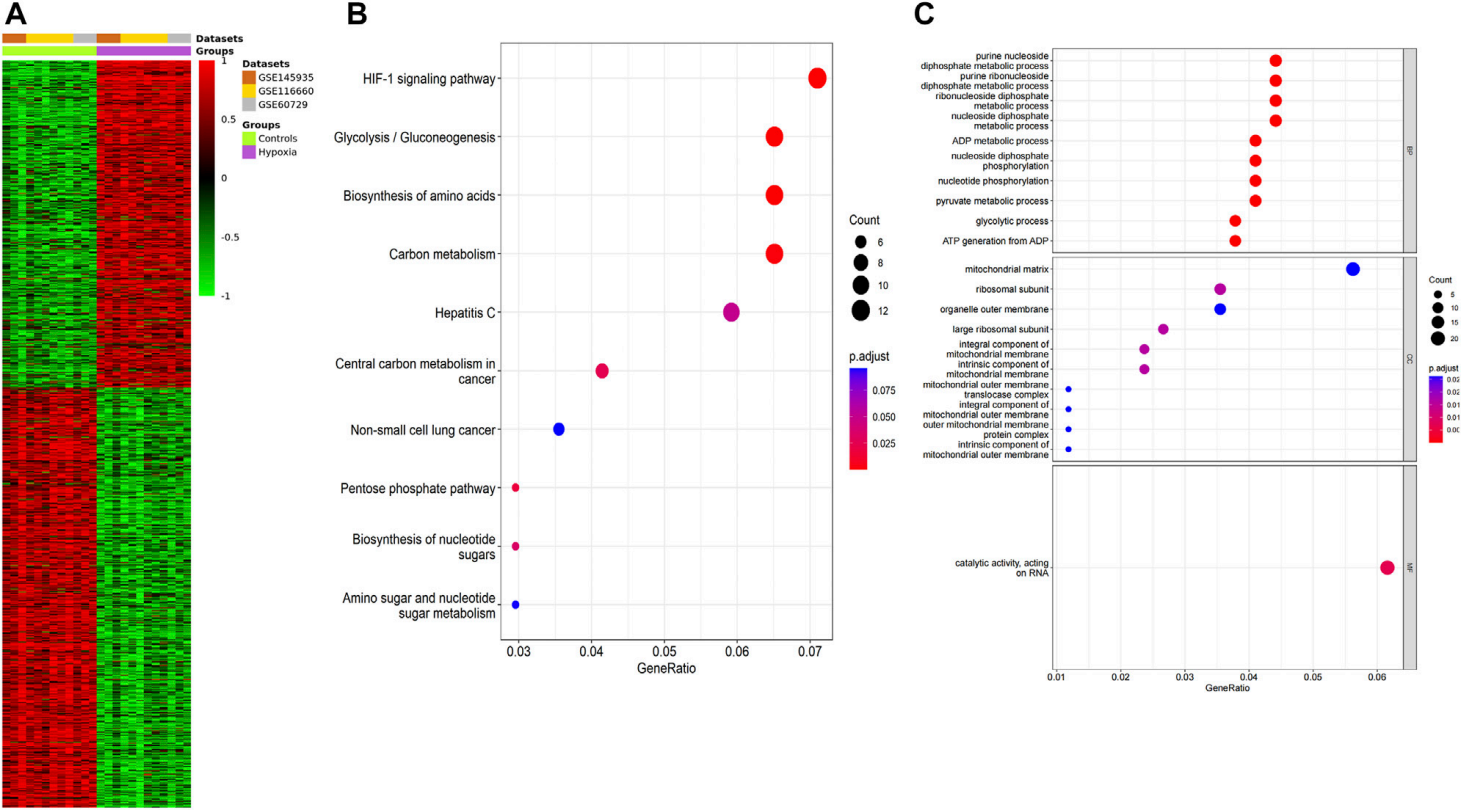
Function annotation of meta-analysis results. **(A)** The heatmap of the DEGs according to the adjusted *p*-value and logFC. Red indicates higher gene expression, and green shows lower gene expression. **(B)** KEGG terms in the enrichment analysis of the DEGs. **(C)** GO terms in the enrichment analysis of the DEGs.

We performed a quality assessment of the three datasets for meta-analysis by drawing PCA plots and a density plot (Figures 3B–D). After quality control, the result of the meta-analysis shows 358 significant genes (209 genes upregulated, 149 genes downregulated) (Supplementary File S5). Next, a heatmap was used to visualize these DEGs, shown in Figure 4A.

### 3.2 Enrichment analysis

GO analysis and KEGG pathway analysis were carried out using the R software. The top 8 biological processes were selected based on P. adjust < 0.05 (Table 1) and were drawn in a bubble plot (Figure 4B). KEGG pathway enrichment analysis showed that DEGs were enriched in the HIF-1 signaling pathway, Glycolysis/Gluconeogenesis, Biosynthesis of amino acids, and Carbon metabolism. GO enriched in enrichment analyses showed that DEGs were mainly enriched in these biological processes: ncRNA metabolic process, nucleotide metabolic process, ATP metabolic process, response to oxygen levels, glycolytic process, cellular response to hypoxia, et al. (Supplementary File S6; Figure 4C). Accordingly, GO and KEGG enrichment analyses both showed that DEGs were mainly enriched in these biological processes and pathways which are closely related to hypoxia acclimatization at high altitudes.

**TABLE 1:** KEGG pathways of meta-analysis results.

ID	Description	Gene ratio	Bg ratio	p.adjust
hsa04066	HIF-1 signaling pathway	12/169	109/8,115	<0.001
hsa00010	Glycolysis/Gluconeogenesis	11/169	67/8,115	<0.001
hsa01230	Biosynthesis of amino acids	11/169	75/8,115	<0.001
hsa01200	Carbon metabolism	11/169	115/8,115	0.0016
hsa05230	Central carbon metabolism in cancer	7/169	70/8,115	0.025
hsa00030	Pentose phosphate pathway	5/169	30/8,115	0.018
hsa01250	Biosynthesis of nucleotide sugars	5/169	37/8,115	0.034

### 3.3 Common DEGs of meta-analysis and GSE46480

**FIGURE 5:**
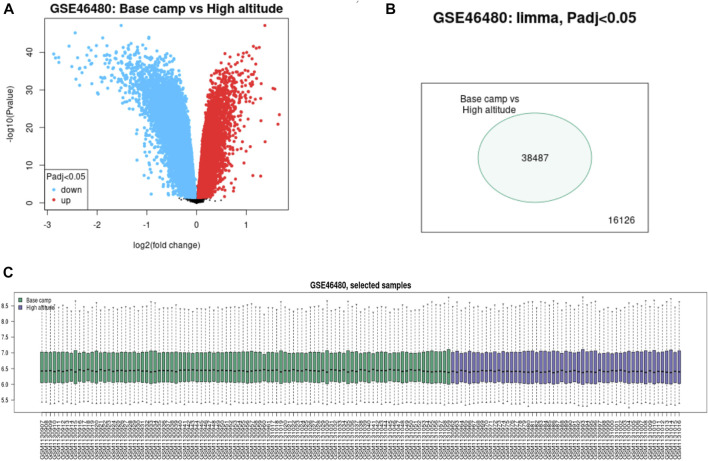
Difference expression of genes analysis of the samples from base camp to high altitude in GSE46480. **(A)** Volcano plot of the DEGs. Blue points represent downregulated genes. Red points represent the upregulated genes. **(B)** Venn diagram of the differentially expressed probe in GSE46480. **(C)** Quality control of the samples of GSE46480.

**FIGURE 6:**
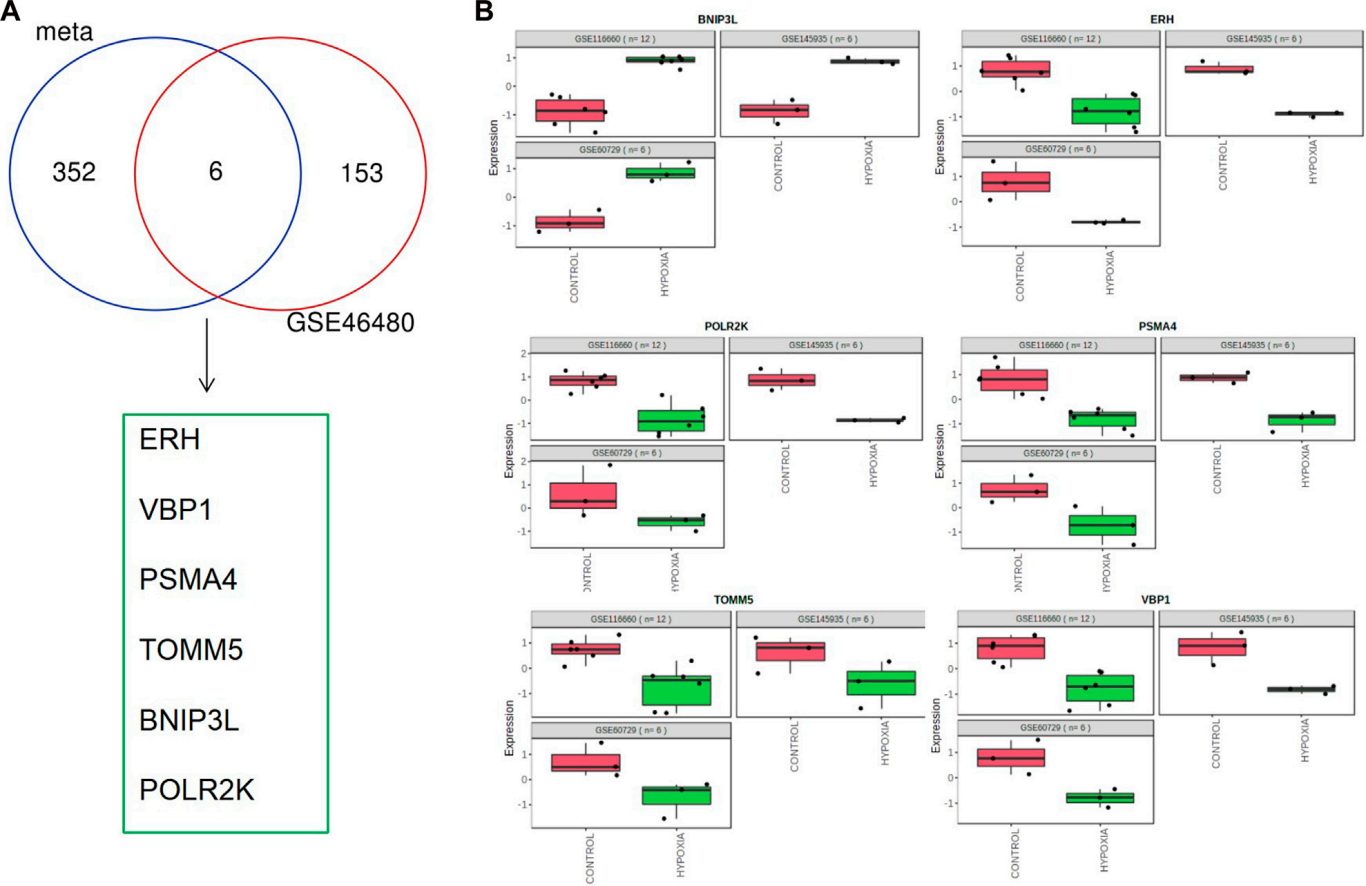
Common differentially expressed genes in meta-analysis and GSE46480. **(A)** Venn diagram of differentially expressed genes identified from the meta-analysis and GSE46480. **(B)** Scatter plots of common differentially expressed genes in the three GEO datasets for meta-analysis.

We download GSE46480 in the GEO database to verify whether the DEGs of the meta-analysis are statistically significant in the process of high-altitude acclimatization and find the relationship between them. 173 DEGs (172 genes downregulated, one gene upregulated) were found between healthy samples at sea level and after acute exposure to altitude in GSE46480 (Supplementary File S7, Figures 5A, B). The data went through rigorous quality control and standardization (Figure 5C). Taking intersections of the DEGs between meta-analysis and GSE46480, we identified six genes associated with high-altitude hypoxia, ERH, VBP1, BINP3L, TOMM5, PSMA4, POLR2K, shown in Figure 6; Supplementary File S8. Except that BINP3L was upregulation, the other five genes showed downregulation in response to hypoxia. And we found that the six genes were all downregulation in GSE46480, which has common trends in gene expression except for BINP3L (Table 2).

**TABLE 2:** Validation of the six differentially expressed genes in GSE46480.

Gene	adj.P.Val	t	logFC	Gene.title
ERH	2.76E-40	−18.01	−1.15	enhancer of rudimentary homolog (*Drosophila*)
VBP1	1.01E-35	−16.24	−1.12	VHL binding protein 1
PSMA4	2.41E-33	−15.39	−1.32	proteasome subunit alpha 4
TOMM5	1.20E-37	−16.94	−1.04	translocase of outer mitochondrial membrane 5
BNIP3L	1.67E-32	−15.09	−1.27	BCL2 interacting protein 3 like
POLR2K	2.83E-38	−17.19	−1.25	RNA polymerase II subunit K

### 3.4 Immune infiltration analyses

**FIGURE 7:**
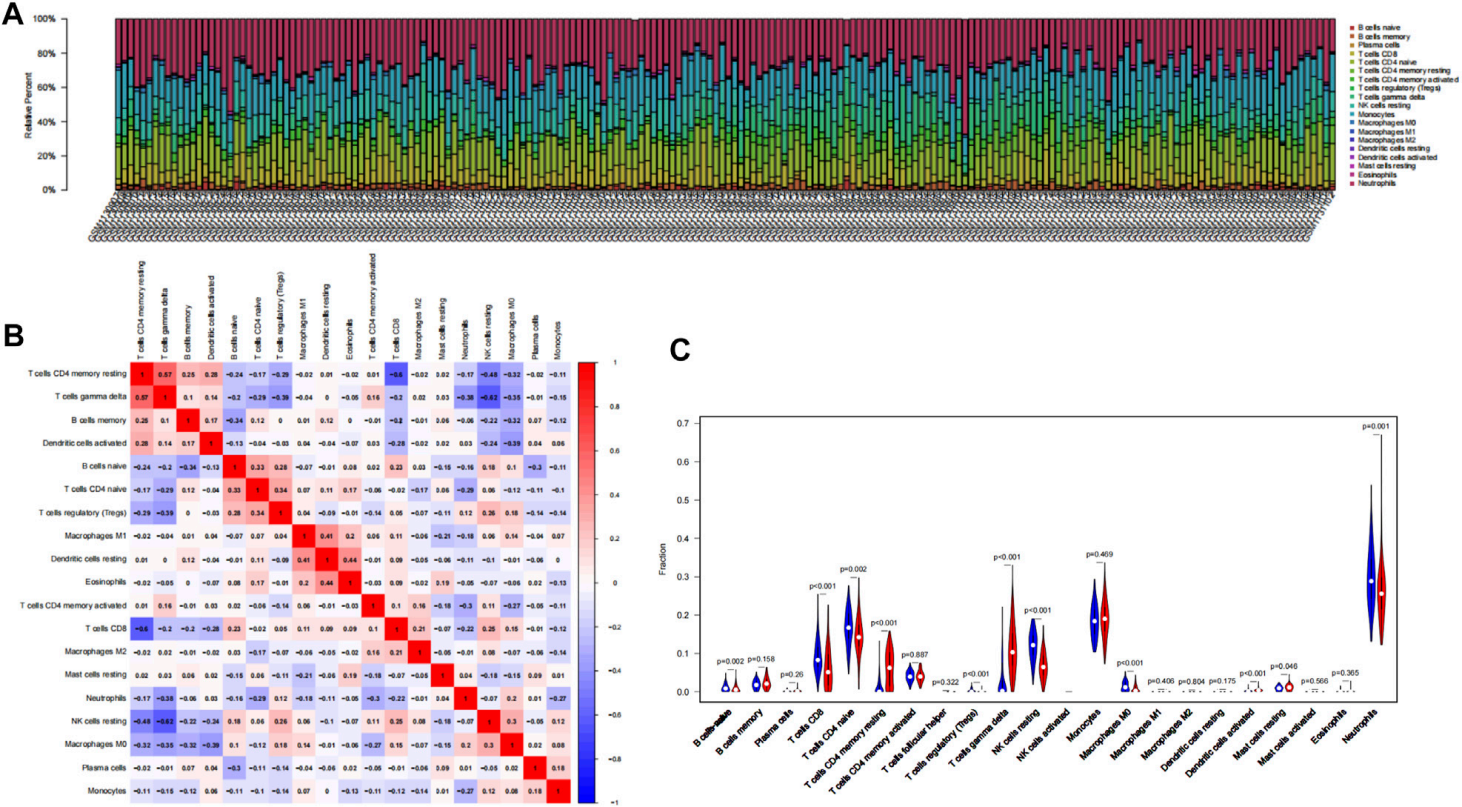
Results of CIBERSORT analysis of GSE46480. **(A)** Landscape of immune cell infiltration. **(B)** Correlation matrix of infiltration degree of immune cells in samples at high altitude. Red indicates trends consistent with the positive correlation, and blue indicates trends consistent with the negative correlation between two immune cells. The bigger the statistics of the number data, the more positive or negative correlation. **(C)** Violin diagram of immune cell proportions in two groups. The blue fusiform fractions on the left represent the basecamp group, and the red fusiform fractions on the right represent the high-altitude group.

To explore whether these six genes are involved in immune regulation in hypoxia acclimatization at high altitudes, we analyzed the role of immune infiltrates between healthy samples at sea level and after acute exposure to altitude in GSE46480. A CIBERSORT analysis can reveal innate and adaptive immune responses during high-altitude acclimatization. Nineteen types of immune cells were detected in at least one sample among 22 types (Figure 7A; Supplementary File S9). The correlation heatmap result showed that T cells CD8 and T cells CD4 memory activated had a negative correlation (*p*-value = −0.60), NK cells resting and T cells gamma delta had a negative correlation, too (*p*-value = −0.62), T cells gamma delta and T cells CD4 memory resting had a positive correlation (*p*-value = 0.57), shown in Figure 7B; Supplementary File S10. The heatmap showed the relative percent of the 22 immune cells (Supplementary File S11). Moreover, the violin plot of the immune cell showed that B cells naive, T cells CD8, T cells CD4 memory naive, T cells regulatory (Tregs), NK cells resting, Macrophages M0, Neutrophils were statistically more in samples in the baseline. T cells CD4 memory resting, T cells gamma delta, Dendritic cells activated, and Mast cells resting statistically more in high-altitude samples (*p* < 0.05), shown in Figure 7C.

**FIGURE 8:**
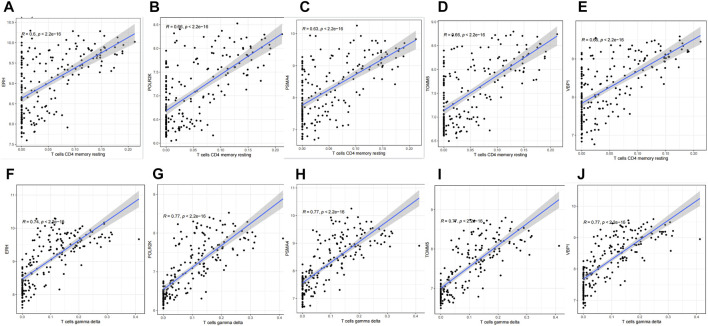
Correlation between hub genes and immune cells infiltration. **(A)** ERH & T cell CD4 memory resting. **(B)** POLR2K & T cell CD4 memory resting. **(C)** PSMA4 & T cell CD4 memory resting. **(D)** TOMM5 & T cell CD4 memory resting. **(E)** VBP1 & T cell CD4 memory resting. **(F)** ERH & T cell gamma delta. **(G)** POLR2K & T cell gamma delta. **(H)** PSMA4 & T cell gamma delta. **(I)** TOMM5 & T cell gamma delta. **(J)** VBP1 & T cell gamma delta.

In the present study, we sought to determine whether the six genes we identified play a role in immune cells. Therefore, scatter plots were performed to survey the correlation between the six hub genes and immune cells. The results showed that except for BNIP3L, all the other five genes have positive correlations with T cells gamma delta and T cells CD4 memory resting according to the screening criteria (R > 0.6, *p* < 0.05), shown in Figure 8.

### 3.5 Verification of the mRNA expression of six hub genes

**FIGURE 9:**
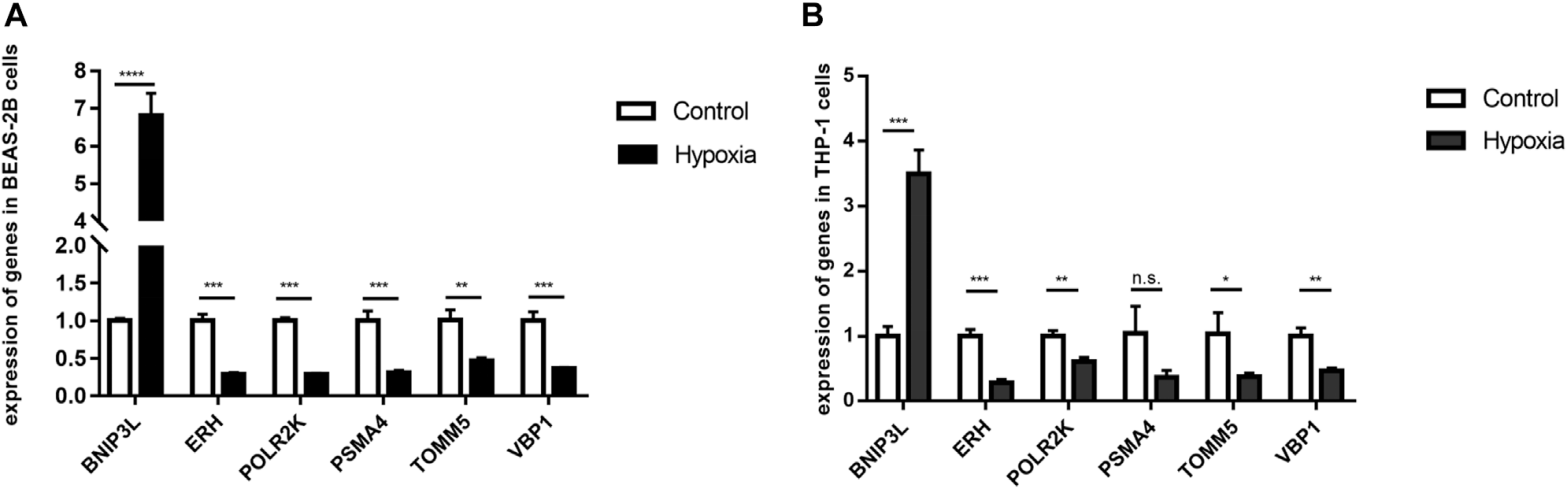
mRNA levels of six hub genes were different in control and hypoxia groups by verified by qRT-PCR results. *p* < 0.05 (*), *p* < 0.01 (**), *p* < 0.001 (***) and *p* < 0.0001 (****) were considered significant. **(A)** BEAS-2B cells. **(B)** THP 1 cells.

qRT-PCR was performed to examine the statistical difference in the mRNA expression of hub genes under two different culture conditions in BEAS-2B cells and THP 1 cells. The two experimental results showed that BINP3L was upregulated, and the other five genes were all downregulated in hypoxia groups, which was consistent with the results from the meta-analysis by bioinformatics methods as shown in Figure 9. Although the expression of PSMA4 in THP1 cells was not statistically different in the two groups, the *p*-value was 0.0513. The experimental results further showed that our meta-analysis is of great significance and has practical value.

## 4 Discussion

Hypoxia affects the development and function of the immune system (Yang et al., 2009). In particular, acute hypoxia-mediated impairment of immune function has been widely described and associated with increased susceptibility to disease (Yi et al., 2020). High-altitude hypoxia could cause an alteration in immunity in individuals with poor acclimatization (Khanna et al., 2018). A large population resides permanently in high-altitude regions like the Tibetan plateau, and every year thousands of people visit the Tibetan plateau for its beauty, therefore, the potential harm of the plateau environment on human health cannot be neglected. Hence, as stated in the introduction, research on the plasticity mechanism of immune function and clarifying their respective roles regarding altitude acclimatization is of great significance. Our study explored common genes and pathways differentially expressed in multiple cell types under hypoxia in an attempt to find the impact of key genes on immune function at high altitudes.

### 4.1 The pathways obtained by meta-analysis are consistent with that in high-altitude acclimatization

GSE145935, GSE116660, and GSE60729 were selected for meta-analysis in this study. What they have in common is that they are all cultured *in vitro* on human cells under hypoxic and normoxic conditions and sequenced to analyze the effect of transcriptome differences on immune function. The difference is that the cell type used in GSE145935 is astrocytes, GSE116660 is NK cells and GSE60729 is DC cells. The 358 DEGs in the meta-analysis confirmed that they had been specifically involved in the HIF-1 signaling pathway, Glycolysis/Gluconeogenesis, Biosynthesis of amino acids, Carbon metabolism, Central carbon metabolism in cancer, Pentose phosphate pathway, Biosynthesis of nucleotide sugars. Interestingly, these signaling pathways are important topics in high-altitude medicine, especially the HIF-1 signaling pathway, Glycolysis/Gluconeogenesis, and Biosynthesis of amino acids and Carbon metabolism. According to the research results, we justified the design of the meta-analysis on the datasets of hypoxia and immune, which could determine important features, patterns, functions, and connections, thus leading to the generation of new biological hypotheses.

The HIF-1 signaling pathway mediated a variety of important processes in hypoxia adaptation, which included Extracellular oxygen sensing (Tanaka et al., 2017), angiogenesis (Fu et al., 2018), and cell proliferation (Yoo et al., 2011), glucose metabolism (He et al., 2018). The HIF protein consists of a constitutively expressed beta subunit and an oxygen-sensitive alpha subunit (Biddlestone et al., 2015). The HIF complex can regulate several hundred genes by binding to hypoxia response elements in gene promoters to increase oxygen supply to hypoxic tissues (Semenza, 2009).

Moreover, HIF-1α is one of the most important regulators of glucose metabolism and has under its control the majority of pathways that can be fueled by glucose, including glycolysis, pentose phosphate pathway, serine synthesis, nucleotide synthesis, and one-carbon metabolism (He et al., 2018). These findings are surprisingly consistent with our KEGG analysis results, which indicates that our analysis is correct and meaningful. Glycolysis/gluconeogenesis is an important biological process in energy metabolism. It has been reported that Tibetans living at high altitudes may be prone to glucose tolerance (Okumiya et al., 2016). The levels of blood insulin and glucose in the Tibetan high-altitude population are lower (Murray, 2016). In addition, glucose uptake increased after insulin stimulation, and skeletal muscle underwent morphological changes due to hypoxia acclimation at high altitudes (Hennis et al., 2015). The glycolysis/gluconeogenesis signaling pathway may play an important role in the high-altitude adaptation of Tibetans living at high altitudes and protect them from the disease.

It has previously been reported that severe environmental hypoxia inhibits insulin/mTOR signaling and amino acid transport in primary human trophoblast cells (Nelson et al., 2003) and in the placenta of pregnant mice *in vivo* (Higgins et al., 2016). Inhibition of amino acid synthesis will reduce the availability of amino acids and may also affect the rate of protein synthesis (Yang et al., 2018). Hypoxia and ER stress generally prevents the Biosynthesis of amino acids (Vaughan et al., 2020), and therefore, it may be an important pathway of high-altitude acclimation.

Under hypoxia physiological conditions, the body will also generate a broad range of biological processes, particularly participation in stress and defense responses, carbon metabolism, protein synthesis, and various metabolic processes. Carbon metabolism supports multiple physiological processes, including redox defense, amino acid homeostasis, Biosynthesis (purines and thymidine), and epigenetic maintenance (Ducker and Rabinowitz, 2017).

Central carbon metabolism (CCM) traditionally includes the glycolysis pathway (EMP), pentose phosphate pathway (PPP), and tricarboxylic acid cycle (TCA). Aside from providing energy, central carbon metabolism also provides precursors to other metabolisms in the body (Bayram et al., 2020). A pentose phosphate pathway initiates the oxidative decomposition of glucose through direct oxidation and can be oxidized and decomposed separately. In addition, it is the main pathway for the metabolism of pentose. In this way, it can enhance the body’s adaptability by complementing and cooperating with EMP and TCA (Minamoto et al., 2015). A redox reaction is one of the main chemical reactions, and oxidoreductase is the enzyme responsible for catalyzing these reactions. Oxidoreductase plays a key role in metabolic pathways such as the tricarboxylic acid cycle and pentose phosphate pathway. Glucose is the most important energy material. It plays the physiological functions of oxidative energy supply (main part), providing a carbon source for synthesizing lipids and proteins and forming glycolipids. Its decomposition pathway in organisms is mainly central carbon metabolism (Ju et al., 2017). When glucose metabolism is abnormal in hypoxic conditions in high-altitude areas, it can cause various body diseases.

Nucleotide sugars are very important for organisms. They are a key link to the Biosynthesis of carbohydrates and their conjugates (Mikkola, 2020). Nucleotide sugar is a glycosyl donor in glycan synthesis, which participates in the correct function and survival of organisms. Glycans mediate the communication and interactions of cells, and defects in glycosylation can cause severe disease, which can lead to fatal outcomes (Endo, 2019).

Hence, hypoxia has wide-ranging effects, causing various biological processes, as we mentioned above. The results of DEGs enrichment analysis of meta-analysis showed that these signaling pathways were closely associated with high-altitude acclimatization. It further shows that it is reasonable to use meta-analysis to find the candidate genes and pathways related to altitude acclimatization.

### 4.2 The six hub genes are closely related to high-altitude acclimatization

The main objective of our study is to identify key genes that have an impact on immune function during altitude hypoxia. Therefore, we selected the dataset GSE46480, which is most relevant to high-altitude disease, for further analysis. The GSE46480 dataset included 98 subjects with acute mountain sickness who participated in the United States Antarctic Program. Sixty-five men and thirty-three women, ranging in age from 26 to 50, were transported from sea level to an altitude of 3,200 m in less than 4 hours. Blood samples were collected from subjects 3 days after arrival at high altitude and peripheral blood mononuclear cells were isolated for sequencing. The 196 samples in the GSE46480 dataset are blood samples collected from 98 subjects at two time points. Moreover, 98 subjects were chosen from healthy volunteers who were diagnosed with acute mountain sickness. Hence, the selection of GSE46480 and the combination of its data helped us to further explore whether the results of the meta-analysis had a meaningful impact on the development of high-altitude disease. We intersected 173 differential genes from GSE46480 with 358 genes from the meta-analysis and finally identified 6 key genes, ERH, VBP1, BINP3L, TOMM5, PSMA4, and POLR2K. As far as we know, little research has investigated the association between these genes and high-altitude hypoxia. Next, we will discuss the role of these genes in the process of high-altitude acclimatization.

ERH (mRNA splicing and mitosis factor) encodes a highly conserved homodimeric protein found in unicellular eukaryotes, plants, and metazoan (Weng and Luo, 2013). A wide range of cellular processes may be affected by eukaryotic ERH proteins, including cell cycle progression, mRNA transcription, and splicing, as well as DNA replication and repair (Tsubota and Phillips, 2016). DNA replication is impaired without ERH, resulting in lower restart rates from a replication stress challenge and sustained DNA damage (Kavanaugh et al., 2015). Also, the ERH gene was continuously regulated during erythropoiesis, and its expression increased during differentiation (da Cunha et al., 2010). When the oxygen partial pressure of tissue decreases and the mucopolysaccharide in the matrix tends to become acidic, it is not conducive to the differentiation of erythroid cells (Thornton et al., 2020). Therefore, the expression of the ERH gene was inhibited during hypoxia at high altitudes.

VBP1 (Von Hippel-Lindau-binding protein 1) physically interacts with von Hippel-Lindau protein (pVHL), which is crucial for the regulation of HIF-1α in the presence of oxygen (Kim et al., 2018). A study revealed that VBP1 could enhance the stability of the pVHL and act as an adaptor molecule in VHL-promoted ubiquitination and the proteasomal degradation of HIF-1α under normoxic physiological conditions (Xu and Her, 2013). In addition to pVHL, HIF-1α is the most important degrading target. Nevertheless, the hydroxylation of HIF-1α occurs less during hypoxia, which leads to HIF-1α accumulation. The accumulated HIF-1α activated the transcription of HIF-1α downstream factors, triggering a continuous transcription of hypoxia response genes (Cockman et al., 2000). Consequently, we can speculate that VBP1 may affect the pVHL-mediated degradation of HIF-1α in high-altitude acclimatization.

BNIP3L (BCL2 interacting protein 3-like)protein localized on the mitochondrial outer membrane is a specific receptor for mitophagy recognition during red blood cell maturation, hypoxia, and metabolic stress (Brennan et al., 2018). Under hypoxic conditions, BNIP3L is critical for hypoxia-induced autophagy and cell survival. BNIP3L may be an important mitochondrial sensor for various stress stimuli, and it promotes mitochondria removal during reticulocyte differentiation (Di Rita et al., 2018). In addition, hypoxia-induced autophagy *via* BNIP3 (BCL2 interacting protein 3) or BNIP3L was shown to be protective against cell death, and the expression of BNIP3 is strongly activated by hypoxia, which is the direct transcriptional target for HIF-1α and inhibits the proliferation of alveolar epithelial cells and promotes their apoptosis (Vara-Perez et al., 2021). Therefore, our meta-analysis showed that the expression of BNIP3L was upregulated in the hypoxia groups in all three studies, which was verified with qRT-PCR.

The TOMM5 subunit is one of three smaller components of TOMM40, which is part of the TOMM complex, translocase of the outer mitochondrial membrane. The protein translocase is located in the mitochondrial outer membrane that transports mitochondrial pre-proteins into mitochondria. There is evidence that TOMM5 helps maintain the structural integrity of the TOMM complex (Kato and Mihara, 2008). Through its association with the TOMM40 precursor, TOMM5 also contributes to the final assembly and placement of the mature TOMM40 in the mitochondrial membrane (Becker et al., 2010). Mice lacking the outer mitochondrial membrane homolog 5 [Tomm5 (−/−)] exhibited an unexpected lung-specific phenotype characterized by extensive intra-alveolar fibrosis, indicating that TOMM5 activity is particularly significant for mitochondrial function in the unique environment of the lung (Vogel et al., 2013). According to their findings in this mouse model, defective TOMM5 alleles could contribute to an increased susceptibility to certain lung diseases in humans and animals (Vogel et al., 2013). Therefore, we supposed that severe hypoxia causes alveolar injury, and the expression of TOMM5 is downregulated in the hypoxic environment presented by high altitude, which is consistent with the results of this study.

PSMA4 (proteasome 20S subunit alpha 4) encodes a subunit of the proteasome (O'Brien et al., 2018). And PSMA4 plays a role in promoting cancer cell proliferation, including proliferation and apoptosis (Zhao et al., 2017). Compared to normal lung tissues, lung tumors have higher PSMA4 mRNA levels, and downregulation of PSMA4 expression reduces proteasome activity and induces apoptosis in lung cancer cells (Deshpande et al., 2013). *In vitro*, PSMA4 knockdown reduces proteasome activity and causes ubiquitinated protein accumulation. The proteasome regulates many cellular processes, including transcription, cell cycle progression, and apoptosis (Wang et al., 2015). One study also found that PSMA family genes were positively correlated with the cell cycle, ubiquinone metabolism, oxidative stress, and immune response signaling. PSMA4 gene is rarely reported in the research of high-altitude medicine. Still, according to the above description, we speculate that it plays an important role in the process of high-altitude acclimatization.

POLR2K (RNA polymerase II, I, and III subunit K) encodes one of the smallest subunits of RNA polymerase II, the polymerase responsible for synthesizing messenger RNA in eukaryotes. Two other DNA-directed RNA polymerases share this subunit (Lin et al., 2012). POLR2K contributes to the Pol III preinitiation complex assembly as an important gene in the RNA processing machinery. Upregulation of POLR2K may facilitate Pol III assembly, which is required for protein synthesis, contributing to cell proliferation and cancer development. Upregulation of POLR2K may facilitate Pol III assembly, contributing to cell proliferation and cancer development (Gutierrez-Arcelus et al., 2019). In a previous study investigating the pathogenesis of pneumonia, POLR2K was found to encode crucial proteins in the PPI network (Huang et al., 2017). However, no studies have been performed to investigate the association between POLR2K and high-altitude acclimatization. In the present study, POLR2K was downregulated in the meta-analysis and the samples at the high altitude of GSE46480.

### 4.3 Immune infiltration analysis shows significant differences in samples at sea level and after acute exposure to altitude

Many diseases are immune-related, including hypoimmune, hyperimmune, or immune dysfunction. Hypoxia induces angiogenesis, reshapes cell metabolism, and regulates the expression of several immunoregulatory molecules. Our question is whether and how the immune function will change when people are exposed to hypoxia at high altitudes. Therefore, we used CIBERSORT analysis to explore the immune cell infiltration in the organism and the relationship between immune cells and 6 key genes during hypoxia at high altitudes. The result showed that the infiltration of several immune cells (T cells CD4 memory resting, T cells gamma delta, Dendritic cells activated, Mast cells resting) was significantly increased, and the infiltration of several immune cells (B cells naive, T cells CD8, T cells CD4 memory naive, T cells regulatory (Tregs), NK cells resting, Macrophages M0, Neutrophils) were significantly decreased in the samples in high-altitude compared to the samples in sea-level. In the correlation analysis of the infiltration degree of immune cells, T cells CD8 and T cells CD4 memory activated, NK cells resting and T cells gamma delta had a negative correlation, while T cells gamma delta and T cells CD4 memory resting had a positive correlation. A hypoxic environment increases inflammation to expedite pathogen clearance by modulating the immune response (Dominguez-Gutierrez et al., 2014). Angiogenesis, metabolism rewiring, and immunomodulatory molecules are all activated by hypoxia (Lin et al., 2020). Considering that the subjects in the GSE46480 dataset developed acute high altitude sickness after arrival at high altitude, and immune infiltration analysis showed significant differences, we think that the oxygen-deprived environment at high altitudes will, therefore, alter the functionality of infiltrated immune cell populations. The infiltrating immune cells may be exerting their deleterious effects in the body after exposure to high altitude; alternatively, infiltration of immune cells into systemic blood vessels or other organs may mediate the deleterious effects of immune cells in high-altitude sickness. Meanwhile, several immune-related genes have been shown to correlate with disease susceptibility. Compared with sojourners from the lowlands, the Tibetan population has evolved distinct high-altitude adaptive features in their long-term habitation in the Tibetan Plateau, particularly the superior resistance to hypoxia. However, the mechanisms of high-altitude adaptation in different human populations are distinct and complex. All subjects in GSE46480 were of high-altitude non-adapted ancestry and they suffered from acute mountain sickness during their initial visit to high altitudes. The inhibition of these genes may contribute to immune cells exhaustion or death, impair immune function, and increase susceptibility to high-altitude sickness. The hub genes in this study strongly correlate with immune infiltrating cells, so they may play an important role in regulating the function of immune cells of lowlanders going to high altitudes for the first time.

In general, ERH, VBP1, PSMA4, TOMM5, BNIP3L, and POLR2K have been identified as the key genes that were associated with immune cells in high-altitude hypoxia. HIF-1 signaling pathway, Glycolysis/Gluconeogenesis, and Biosynthesis of amino acids and Carbon metabolism were important signaling pathways that occur in cells to adapt to hypoxic environments. We apply a meta-analysis to harmonize a deliberately varied selection of gene expression datasets of different cell types. Our findings are consistent with the three datasets included in the meta-analysis in demonstrating that hypoxia affects the immune function of the body and that key genes play an important role in the hypoxic process. Furthermore, our study identified six key genes that are strongly associated with hypoxia at high altitudes and verified that they play important roles in multiple types of cells and influence immune function. These results may provide a new perspective for the study of high-altitude acclimatization. However, this study still has some limitations. First, the results of this study were only validated with qRT-PCR experiments, and in-depth experiments will be conducted in future studies. Second, our meta-analysis had small sample sizes, and thus the results ought to be interpreted with caution.

## 5 Conclusion

The 6 hub genes, pathways, and immune infiltration analysis we identified may be involved in high-altitude acclimatization. These findings will help us to enhance our general understanding of the molecular mechanism of immune function during high-altitude hypoxia. Targeting hypoxia-sensitive pathways in immune cells is of interest in treating high-altitude sickness. They are involved in processes that are targets for drug development, which will guide precision medicine treatment. This study provides support for further research on high-altitude acclimatization.

## Data Availability

Publicly available datasets were analyzed in this study. This data can be found here: https://www.ncbi.nlm.nih.gov/.
