# Supplementary figures and images for: Identification of key pathways, genes and immune cell infiltration in hypoxia of high-altitude acclimatization via meta-analysis and integrated bioinformatics analysis
Source: Front Genet. 2023 Mar 24;14:1055372. doi: 10.3389/fgene.2023.1055372 (PMC10080023; doi:10.3389/fgene.2023.1055372)

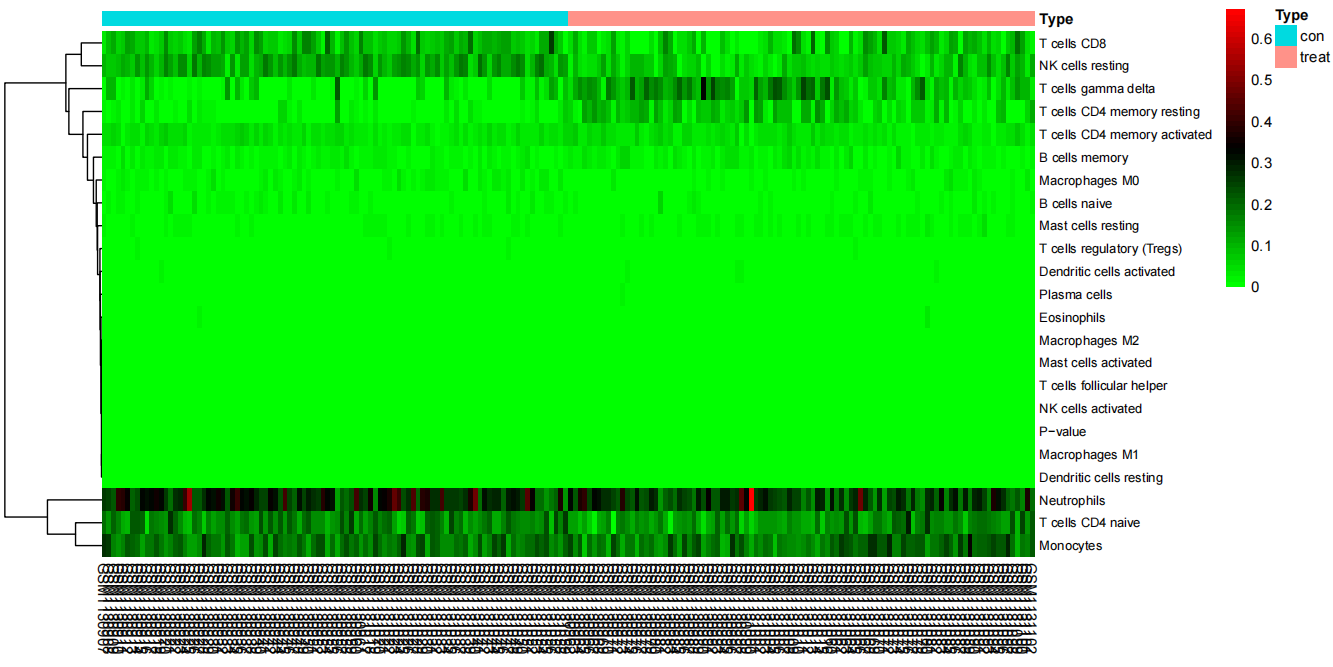

Supplement: Supplementary file 5 [file Image1.TIF]
